# Qualitative Characteristics of Semolina–Pulse Flour Mixes and Related Breads

**DOI:** 10.3390/foods14213720

**Published:** 2025-10-30

**Authors:** Michele Canale, Rosalia Sanfilippo, Salvatore Moscaritolo, Maria Carola Fiore, Maria Concetta Strano, Maria Allegra, Giancarlo Fascella, Giovanni Gugliuzza, Alfio Spina

**Affiliations:** 1 Council for Agricultural Research and Economics (CREA), Research Centre for Cereal and Industrial Crops, Corso Savoia, 190, 95024 Acireale, Italy; rosalia.sanfilippo@crea.gov.it; 2 Council for Agricultural Research and Economics (CREA), Research Centre for Cereal and Industrial Crops, S.S. 673 km 25.200, 71122 Foggia, Italy; salvatore.moscaritolo@crea.gov.it; 3 Council for Agricultural Research and Economics (CREA), Research Centre for Plant Protection and Certification, c/o Department of Agricultural, Food and Forest Sciences, University of Palermo, Viale delle Scienze, Building 4, 90128 Palermo, Italy; mariacarola.fiore@crea.gov.it (M.C.F.); giancarlo.fascella@crea.gov.it (G.F.); giovanni.gugliuzza@crea.gov.it (G.G.); 4 Council for Agricultural Research and Economics (CREA), Research Centre for Olive, Fruit and Citrus Crops, Corso Savoia, 190, 95024 Acireale, Italy; mariaconcetta.strano@crea.gov.it (M.C.S.); maria.allegra@crea.gov.it (M.A.)

**Keywords:** bread, durum wheat, flour mixes, pulses, technological properties

## Abstract

In recent years, pulse flours have gained attention in baked goods for their nutritional value. This study evaluated the effects of incorporating common bean, yellow pea, and grass pea flours (20%, 30%, 40%) into durum wheat semolina on the technological, physical, and rheological properties of flours, doughs, and breads. Combining pulse flours with durum wheat semolina allows for improved dough handling and processing performance, leveraging the functional properties of both ingredients. Water absorption increased with pulse flour addition (average 1.90 g H_2_O/g dry matter), though higher levels of yellow pea and grass pea reduced it. Color changes were most evident with common bean flour. Leavening rates varied, reaching 144% after 60 min with 30% yellow pea and 68.75% after 40 min with 30% common bean. Rheological results indicated longer dough development and stability times but reduced strength and extensibility, with higher tenacity. Bread volume decreased from 276.25 cm^3^ (control) to 208.75 cm^3^ (40% common bean). Crumb porosity declined, particularly with common bean flour, producing smaller pores. Grass pea flour promoted browning, enhancing color contrast. Texture analysis showed harder, more gum-like breads with higher chew resistance: hardness ranged from 15.85 N (20% common bean) to 30.45 N (40% yellow pea). Gumminess and chewiness increased, while cohesiveness decreased. Overall, pulse flour integration alters bread quality, yet represents a promising approach to creating healthier, functional, baked products.

## 1. Introduction

Climate change, global population growth, sustainability issues, and the fight against food waste are significantly affecting the entire agri-food supply chain. In production planning, it is essential to adopt good practices that promote a sustainable production system, ensuring a future for current and future generations [1,2,3].

Growing consumer interest in health and purchasing nutritious foods has spurred research to address the challenges of meeting the demand for healthier baked goods. To this end, the bakery industry has been adapting its production for several years to create functional and sustainable foods enriched with fibers, plant proteins, and other bioactive compounds [4,5,6], thereby reusing waste and/or producing nutritionally complete products. One example is the use of pulse flour to enrich soft and durum wheat flours to produce bread, pasta, and other baked goods with high added value, sustainability, and functionality [7,8,9,10]. The addition of legumes to the human diet offers various health benefits, including a reduction in the risks of chronic diseases, diabetes, and certain types of cancer [11,12,13].

Pulse flours, such as those from common beans, yellow peas, and grass peas, are rich in proteins and often provide a better balance of essential amino acids, particularly lysine. Additionally, they are excellent sources of dietary fibers, vitamins, and minerals, which enhance the overall nutritional profile of cereal-based products [14,15]. Incorporating pulses into food processing contributes to the formulation of plant-based protein products and promotes food sustainability by decreasing reliance on animal sources and supplying value-added, functional foods. In addition to their nutritional properties, pulse flours provide functional benefits that play a key role in food formulation and processing, owing to their ability to improve water-holding capacity, viscosity, and emulsifying and foaming properties, as well as to contribute to the structure and stability of the final product [16,17,18].

Although durum wheat is primarily known for its role in pasta production, it is also extensively used for bread-making in the Mediterranean region. In several Middle Eastern countries, between 70% and 90% of durum wheat is allocated to bread production. This cereal is particularly valued for its positive technological characteristics, such as high protein content, good water absorption, and the ability to form a strong gluten network, which confer excellent bread structure, a distinct yellow color, and typical odor and flavor. A wide variety of traditional breads are made using durum wheat, including Khobz, in Syria, Lebanon, and Jordan; Baladi and Shami in Egypt; Tannur and Saaj in Syria and Lebanon; Tabouna, Mlaoui and M’Besis in Tunisia; and Tandir Ekmegi, in Turkey [19]. Italy, particularly its southern regions, is also a major producer of durum wheat and is renowned for high-quality bread products such as the Dittaino bread (Pagnotta del Dittaino) and Altamura bread (Pane di Altamura), both protected by the European Commission as PDO (Protected Designation of Origin) [20].

In contrast, replacing wheat flour and/or semolina with pulse flour reduces gluten content, leading to technological challenges in the flour, dough, and final product. These difficulties primarily stem from differences in protein composition, which affect the viscoelastic properties of wheat gluten, including water solubility, primary structure, and size distribution. The inclusion of non-gluten-forming proteins weakens the dough by diluting it. Since pulse proteins cannot form gluten networks, they interact weakly with wheat proteins, reducing dough viscoelasticity and impairing air incorporation and gas retention during fermentation. This consequently results in a reduced loaf volume, a denser crumb structure, and more compact bread [21,22,23,24,25].

This study aims to evaluate the effects of incorporating three different pulse flours (common bean, yellow pea, and grass pea) at high percentages (20%, 30%, and 40%) into durum wheat semolina, analyzing the technological, physical, and rheological properties of the resulting doughs and breads. The objective of this research is to provide a comprehensive overview of the impact of high levels of pulse flour integration on durum wheat-based baked products.

## 2. Materials and Methods

### 2.1. Wheat Flours and Origin of Pulses

Durum wheat re-milled semolina (SC) was sourced from the Agricultural Cooperative Society ‘Valle del Dittaino’ a.r.l. in Assoro (Enna, Italy) (latitude 37°57.17′ N; longitude 14°44.87′ E). The majority of the particles in the re-milled semolina used measured between 160 and 200 µm (Table 1).

**Figure 1:**
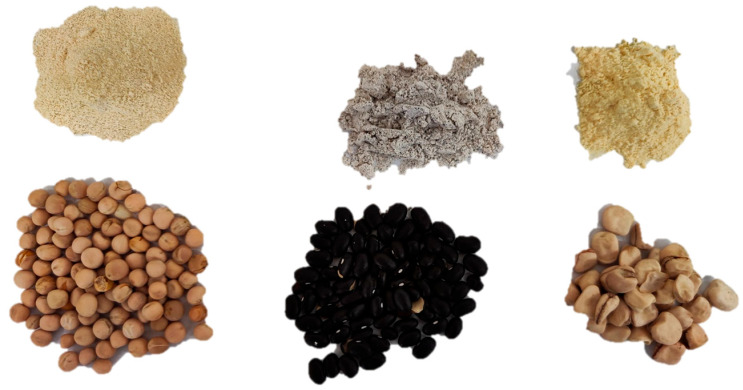
Seeds and flours of pulses (from sx to dx: yellow pea, common bean, and grass pea).

Yellow pea (YP) (*Pisum sativum* L. subsp. *arvense*) cv ‘Orchestra’ and grass pea (GP) (*Lathyrus sativus* L.) ‘Licodia Eubea’ landrace (Figure 1) were grown during the 2023/24 season in the farm ‘Amico’, located in Caltanissetta, Italy (latitude: 37°25′18″ N, longitude: 14°1′4″ E, 350 m a.s.l). The area features regosols soil with medium texture [26], average annual rainfall of 450–500 mm, and average annual temperature of 16 °C (provided by the Sicilian Agrometeorological Information Service—SIAS).

Common bean (CB) (*Phaseolus vulgaris* L.) cv ‘Nanu Niuru’ (Figure 1), a Sicilian local variety characterized by a typical dark tegument and creamy white cotyledons, was kindly provided by the “Living Plants Germplasm Bank” of Ucria (Messina, Italy). It was grown during the 2023/24 growing season at the farm ‘AgriCultura Terra di Santo Stefano’, located in Santo Stefano di Briga, Messina (latitude 38°10.19 N, longitude 15°47.96 E, 300 m a.s.l), characterized by acid brown soils and medium texture [26]. The site has an annual rainfall of 800–900 mm and an average annual temperature of 16 °C (SIAS).

### 2.2. Milling Process and Composition of Mixed Flours with Pulses

The yellow pea and grass pea seeds were milled using an ancient mill equipped with high-hardness French natural millstones from La Ferté-sous-Jouarre (Figure 1). This mill operates at 300 kg/h, enabling a slow grinding process that avoids overheating. Through a pneumatic transport system, the flour was conveyed to a ‘plansichter,’ consisting of frames, sieves, veils, and expellers, which removed the seed coats and refined the flour.

Common bean seeds were ground into whole meal flour using a ‘Waldner Luis’ small stone mill (Lienz, Austria) with a capacity of 8 kg/h. The resulting flour was then refined using a Cyclotec type 120 mill (Falling Number, Huddinge, Sweden) equipped with a 500 µm sieve, in order to obtain common bean flour with a grain size > 35 mesh (Table 1; Figure 1).

Finally, re-milled semolina was blended with each of the pulse flours in three proportions: 20%, 30%, and 40%, in order to prepare the cereal-pulse blends used in the study.

The particle size distribution of the pure and mixed flours are reported in Table 1.

### 2.3. Determination of Moisture, Ash, and Color Characteristics in Flours and Breads

The moisture content in flours and breads was determined according to the AOAC method 935.25 [27], by drying in an oven (Memmert, Milan, Italy) at 103 °C, until a constant weight was achieved. The results were expressed as g/100 g. Ash content was determined according to the ISO method 2171 [28].

The color of the flour, bread crumb, and crust was analyzed with a CR 200 colorimeter (Minolta, Osaka, Japan). The CIELab colorimetric model was adopted by expressing the results according to the coordinates L* (light vs. dark, where a low number (0–50) indicates dark, and a high number (51–100) indicates light), a* (red vs. green, where a positive number indicates red, and a negative number indicates green), and b* (yellow vs. blue, where a positive number indicates yellow, and a negative number indicates blue). To express the darkness of the loaves after baking, the brown index was used, calculated as follows [29]:

Brown Index = 100 − L*

### 2.4. Water- and Oil-Binding Capacity of Flours

Water- (WBC) and oil-binding capacity (OBC) were determined using a modified method described by Sanfilippo et al. (2023) [29].

Following centrifugation at 4200 rpm for 30 min at 20 °C (Heraeus Multifuge X3 FR, Thermo Scientific, Waltham, MA, USA), the solid residue was weighed.

The water-binding (WB) and oil-binding (OB) capacities of the flours were calculated using the following formula:

$$\mathrm{WBC}\;(\mathrm{or}\;\mathrm{OBC})=\frac{100-W_{s}}{100-H_{f}}$$

where

$W_{s}$ = weight of the sample after centrifugation (g)

$H_{f}$ = moisture content of the flour (%).

The water- and oil-binding capacities (WBC and OBC) were measured to evaluate the intrinsic ability of the flour to retain water or oil, reflecting its compositional properties such as fiber and protein content. These measurements provide an indication of the flour’s functional potential in dough and baked products.

### 2.5. Rheological Analyses

Farinographic analyses were conducted using the farinograph (Brabender, Duisburg, Germany), equipped with Farinograph^®^ software, ver. 2.3.7, according to the AACC method 54–21.02 [30]. The water absorption determined by the farinograph corresponds to the amount of water required to reach 500 Brabender Units (BUs), representing a rheological parameter related to dough consistency and handling properties, rather than the intrinsic water-binding capacity of the flour.

An alveograph (Tripette et Renaud, Chopin Technologies, Villeneuve la Garenne, France) equipped with the Alveolink NG V1.04/99 software (Tripette et Renaud, Chopin Technologies, Villeneuve la Garenne, France) was used to determine the dough strength (W) and the tenacity/extensibility ratio (P/L) according to the UNI 10453:1995 [31].

### 2.6. Leavening Test

Samples of mixed flour and re-milled semolina were evaluated for their leavening capacity using the method described by Canale et al. (2022) [32].

Doughs were prepared using re-milled semolina, either pure or partially replaced. Dehydrated yeast (3 g/100 g flour) was dissolved in distilled water at 35 °C in an amount corresponding to the farinograph water absorption at 500 B.U. The mixtures were kneaded for 5 min, and 25 g portions of dough were transferred into lightly oiled 250 mL graduated cylinders. After compacting, the initial volume was recorded. The cylinders were then incubated at 30 ± 2 °C, and the increase in dough volume was measured every 10 min until two consecutive readings showed no further expansion.

### 2.7. Bread Production

The bread was made according to the recipe given in Table 2. The other raw materials used were yeast (Lievital, Lesaffre Italia spa, Parma, Italy), salt (Sosalt spa, Trapani, Italy), and distilled water (by farinograph absorption at 500 B.U.). The doughs were made and baked as described by Canale et al. (2024) [33].

Doughs were mixed in a laboratory mixer at 25 °C for the time required to achieve optimal development, as indicated by the farinograph. After mixing, the dough was fermented in a thermostatic chamber equipped with a steam humidifier at 32–35 °C and 75–80% relative humidity for 90 min. The doughs were then divided, transferred into metal baking tins (7 × 18 × 5 cm), and subjected to a second fermentation under the same conditions for an additional 90 min. Baking was carried out in an electric oven for 7 min at 215 ± 5 °C, followed by 33 min at 165 ± 5 °C.

### 2.8. Physical Properties of Bread and Texture Profile Analyses (TPA)

The volume of the bread was determined using the rapeseed displacement method according to AACC 10-05 [30]. Bread height was measured using a digital caliper (Scienceware^®^ Digi-Max™, Staten Island, NY, USA) and bread weight was recorded using a digital scale (Ohaus, Adventurer Pro AV2102C, Nänikon, Switzerland).

Crumb porosity was assessed by visually comparing the center slice of each loaf bread with eight Dallmann reference images representing different crumb structures [34]. The evaluation followed an eight-point scale, where a score of 1 corresponds to an open and irregular structure with large, uneven cells, and a score of 8 represents a uniform and compact crumb with small, regular cells.

Bread crust hardness was evaluated using a texture analyzer (Zwick/Roell Z 0.5, Ulm, Germany) equipped with a cylindrical stainless-steel flat probe (8 mm diameter) at a test speed of 1 mm/s and an applied deformation of 20% (force shutdown threshold). The breaking force of the crust was expressed in Newtons (N).

Bread texture was further analyzed for elasticity, cohesiveness, gumminess (N), and chewiness (N × mm) on 15 mm thick slices using the Texture Profile Analysis (TPA) test as described by Rózylo et al. (2011) [35], with slight modifications. The TPA test was performed in triplicate using the same texture analyzer equipped with a stainless-steel compression probe (75 mm diameter), applying double compression at 50% and 10% of slice height at a speed of 1 mm/s.

### 2.9. Statistical Analysis

All analyses were carried out in triplicate and the results are presented as means ± standard deviation. Comparison of means was performed using one-way analysis of variance (ANOVA) in Statgraphics^®^ Centurion XVI (Statpoint Technologies, The Plains, VA, USA), and statistical significance between means was determined using the Tukey test (*p* < 0.05).

A principal component analysis (PCA) was performed on the entire dataset, encompassing the physical and chemical characteristics of flours, doughs, and breads obtained with increasing percentages of integration of three pulse flours (common bean, yellow pea, and grass pea). The variables used, grouped into four categories for interpretative clarity, were as follows: flour physicochemical properties (Flour_Dry matter, Ash, WBC, OBC, Flour_L*, Flour_a*, Flour_b*); dough rheological parameters (Dough development time, Stability, Water absorption at 500 B.U., W, P/L); bread physical and textural attributes (Bread volume, Bread height, Bread weight, Bread Moisture, Crumb porosity, Hardness, Springness, Gumminess, Chewiness, Cohesiveness); and bread color characteristics (Crust_Brown index, Crust_a*, Crust_b*, Crumb_Brown index, Crumb_a*, Crumb_b*). PCA was performed on the correlation matrix, which inherently standardizes the variables (mean-centered and scaled to unit variance), ensuring equal weighting regardless of their original measurement units. Principal components were retained according to the Kaiser criterion (eigenvalues > 1) and based on cumulative explained variance exceeding 65%. PCA was conducted using the PAST (PAleontological STatistics) software package (4.04), 2011 [36].

## 3. Results and Discussion

### 3.1. Physical Analyses of Flours

Based on the obtained data (Table 3), ash values were higher for the YP40 flour, reaching 2.87 g/100 g d.m., and lower for the SC control, which recorded 0.79 g/100 g d.m. According to the findings for the YP40 sample, although the values were lower due to the presence of semolina, they are consistent with those reported by other authors for pure dehulled pea seeds, which reached 3.52 g [37]. This variability seems to be influenced by the cultivar x environment (GxE) interaction, which can affect protein and ash content [38]. CB40 and GP40 showed values in line with those found by other authors for pure samples, ranging from 4.60 to 5.00 g for beans [39] and from 2.68 to 3.92 g for grass pea [40]. From a technological perspective, higher ash content can be advantageous as it is often associated with greater mineral richness and may enhance dough coloration; however, it can also negatively affect gluten development and dough handling, potentially reducing bread volume and modifying texture. Moreover, differences in the particle size distribution of the two matrices can further influence water absorption, dough rheology, and the overall structure of the final product, as finer fractions tend to form more cohesive doughs, while coarser particles may disrupt the gluten network.

Regarding the re-milled semolina ash content, the data was slightly lower (0.79 g) than those reported by other authors [41] for ‘PDO Pagnotta del Dittaino’ bread (from 0.86 to 0.89 g).

The colorimetric parameters (Table 3) indicated statistically significant differences among the samples. The L* parameter ranged from 87.79 (GP20) to 83.47 (CB40), a* ranged from −2.50 (GP30) to −0.50 (CB40), and b* values ranged from 20.84 (GP40) to 5.41 (CB40). The differences observed in the individual chromatic coordinates, summarized with ΔE, confirmed these findings. The integration of pulse flours produced progressively greater color differences compared to the control. The inclusion of common bean (CB) flour shifted the a* values toward less negative numbers, indicating a movement toward less green coloration, while yellow pea (YP) and grass pea (GP) flours increased the yellow index of the mixes compared to the control. Similar changes in semolina coloration following supplementation with various pulse flours have been observed by other authors, with comparable trends for substitution rates up to 40% [42].

Water binding capacities (WBC) (Table 3) were higher in CB-enriched mixed flours, with average values of approximately 2.00 g H_2_O/g d.m., driven by the higher absorption capacity influenced by CB40, which recorded 2.19 g H_2_O/g d.m. The increase in WBC is associated with the higher fiber content, which was also because the bean seeds were not dehulled, as observed by other authors [43]. WBC values of mixed flour enriched with common bean flour are consistent with those reported in a previous study [29], where the inclusion of 10% common bean flour significantly increased the WBC values. Integration with YP flour displayed a decrease as the percentage level increased. These findings are consistent with results from other authors using pure, dehulled yellow pea flour [44]. The decrease in WBC with increasing yellow pea flour is likely due to its lower fiber content and reduced water-binding capacity compared to whole bean flour.

Similarly, for grass pea, water absorption values were consistent with other studies on water retention capacity in samples of different particle sizes, with a range from 2.12 to 1.75 g/g [45].

Regarding the OBC variable, the integration of various pulse flours did not lead to statistically significant differences among the samples, with values ranging from 2.37 g oil/g d.m. (GP20) to 2.76 g oil/g d.m. (SC) (Table 3). The SC and the bean flour-enriched samples values are in agreement with previous findings [29], while GP values were higher than those reported by other authors [46] for 100% GP flour (1.51 mL/g), as well as for 100% yellow pea flour (1.90 g/g) [27].

### 3.2. Technological Analysis of Doughs

**Figure 2:**
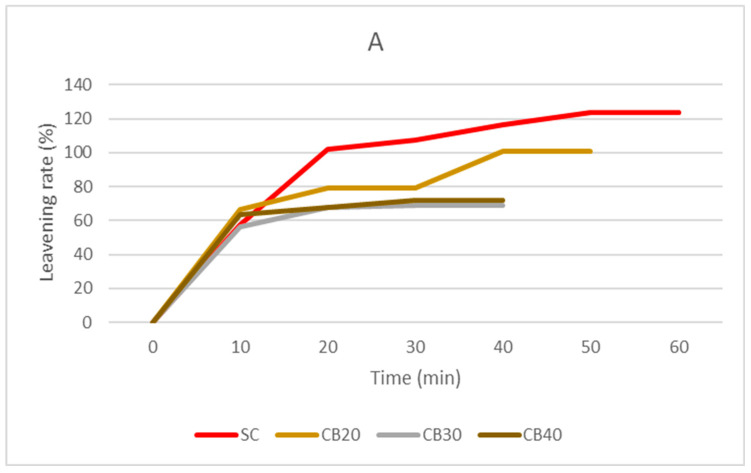

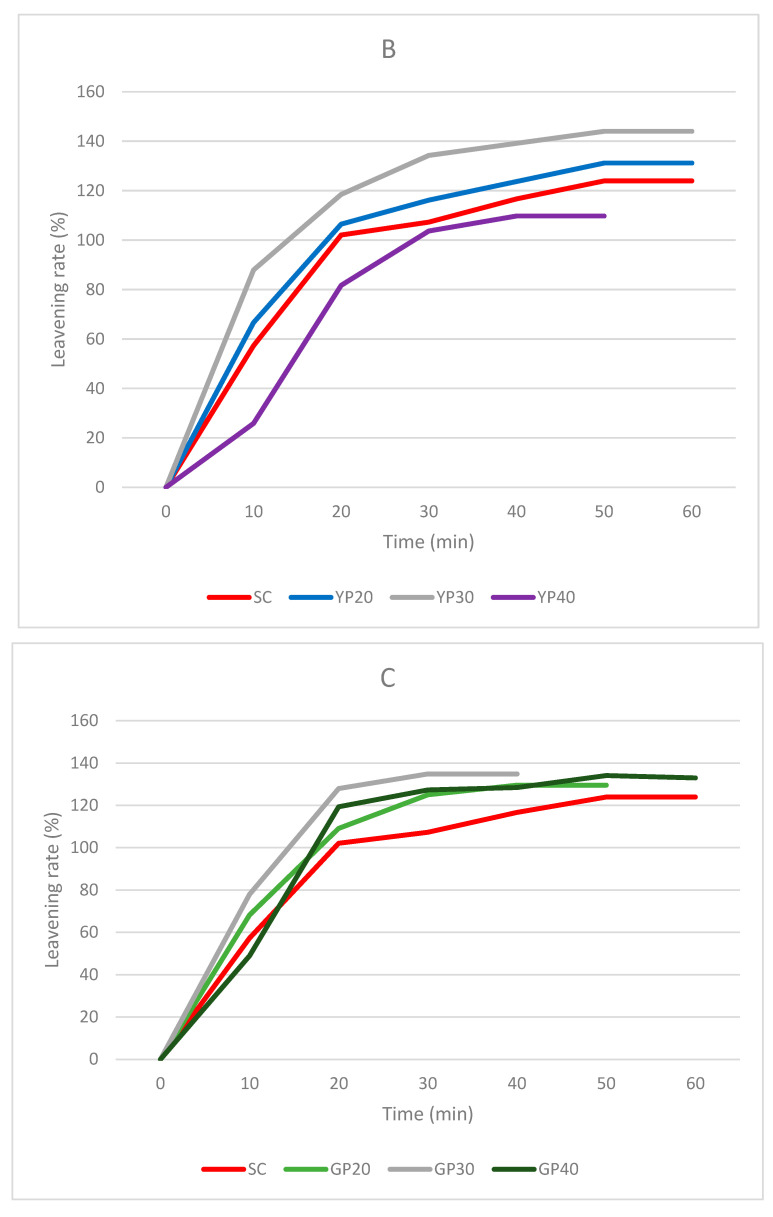
Leavening rate (%) at increasing levels of replacement (20, 30, 40%) of the samples of flours with integration of three pulse flours: common bean (**A**), yellow pea (**B**), and grass pea (**C**). SC: control semolina; CB: semolina with common bean flour at different integration percentages (20/30/40%); YP: semolina with yellow pea flour at different integration percentages (20/30/40%); GP: semolina with grass pea flour at different integration percentages (20/30/40%).

The leavening rates (Figure 2) showed significant variability based on the integration percentages and the legume species used. The range fluctuated between a maximum leavening rate of 144% in 60 min for the YP30 sample and 68.75% in 40 min for CB30. Notably, the samples enriched with common bean flour exhibited the lowest growth rates. Compared to a previous study using a different bean variety with a maximum integration of 10%, the highest leavening rate observed was 138% in 50 min [29]. The reduced fermentative capacity of the common bean flour-enriched samples, compared to those with yellow pea and grass pea flour, may be associated with the higher fiber content, which hinders proper leavening.

As noted by other authors, the presence of fiber tends to obstruct proper dough leavening both by absorbing water necessary for yeast metabolism [47,48] and by limiting the capacity of the gluten network to retain the gases responsible for fermentative growth, due to its reduced elasticity and extensibility, thereby weakening the dough structure.

The addition of pulse flours has also highlighted differences in the technological parameters of the doughs. According to the farinograph data (Table 4), the addition of common bean, yellow pea, and grass pea flours generally increased the dough development time and stability compared to the control. However, the grass pea flour at a concentration of 20% (GP20) showed a development time of 1.70 min, which was lower than SC (1.80 min). Similarly, the yellow pea flour at 40% (YP40) exhibited a stability time of 1.25 min, which was less than SC stability time of 2.70 min. Regarding water absorption (Table 4), no statistically significant differences were observed among all samples, except for those with the addition of 40% GP flour. In particular, all integration rates of CB and YP showed no significant differences between them, while observable differences in water absorption were found for GP, confirming the results presented in Table 3 on water retention capacity.

Studies conducted on different varieties of beans incorporated (15%, 25%, 35%) into soft wheat flour showed similar trends, albeit with slightly higher values for development and water absorption [49]. Similarly, for yellow pea, some studies on 10% incorporation confirmed high values for stability (5.6 min) and water absorption at 500 B.U. of approximately 61% [50].

According to other authors, GP flour showed lower water absorption values in the farinograph compared to CB and YP flours, with values around 55% at 25% incorporation and average development times of 5.7 min [51].

Significant statistical differences were also observed in alveograph results (Table 4) between the legume mixes and the control (SC). Pulse flours had a deteriorative impact, reducing dough strength (W) and tenacity/elasticity ratio (P/L). The W values ranged from 246.00 × 10^−4^ J (SC) to 21.00 × 10^−4^ J (YP40). Meanwhile, the P/L ratio varied from 5.91 in CB40 to 1.19 in GP30. The analysis of W and P/L indicated that excessive tenacity, combined with lower strength, results in doughs unsuitable for producing medium-to-long-fermentation baked goods. For all types of pulse mixes, increasing incorporation resulted in a decrease in W and an increase in the P/L ratio.

The results obtained from incorporating CB flour align with previous studies, which reported W values of approximately 187.0 × 10^−4^ J and 2.84, respectively, for W and P/L in samples with 20% germinated bean flour incorporation [52].

The moisture content of the enriched breads (Table 5) with various pulse flours did not show statistically significant differences, with values ranging from 36.03% in CB20 to 30.75% in GP40. A trend toward lower moisture content was observed for grass pea (GP) breads with increasing substitution levels. This pattern was not consistent for common bean (CB) or yellow pea (YP) breads. The slight reduction in moisture content for some samples could indicate a reduced ability to retain water during baking, likely due to the diminished water-binding capacity of gluten in the presence of higher levels of pulse flour.

**Figure 3:**
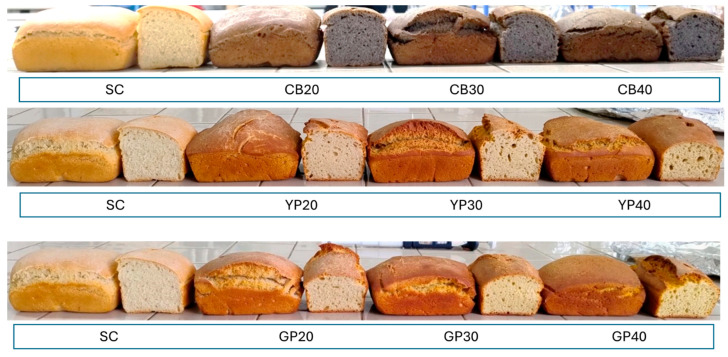
Breads at different levels of replacement with pulse flours. SC: control semolina; CB: semolina with common bean flour at different integration percentages (20/30/40%); YP: semolina with yellow pea flour at different integration percentages (20/30/40%); GP: semolina with grass pea flour at different integration percentages (20/30/40%).

The bread volume decreased (Figure 3) with increasing pulse flour incorporation, with values ranging from 276.25 cm^3^ in SC to 208.75 cm^3^ in CB40. This significant reduction in volume (Table 5) has also been observed by other authors in whole wheat breads fortified with soy flour [53], whole wheat flour fortified with fermented chickpea flour [54], and pea and soy protein isolates [55]. The most noticeable contraction was observed in bean-enriched bread, likely due to the stronger effect of fiber compared to the dehulled protein pea and grass pea samples. Fiber tends to dilute gluten, hindering the dough’s gas retention capacity rather than its gas production, as also reported by other authors [56].

Except for CB20, the YP20 and GP20 breads had slightly higher heights compared to the control (55.25 mm), reaching around 56 mm (Table 5); however, these differences were not statistically significant. No statistically significant differences were observed in the bread weights, which is consistent with results reported by other authors for breads with 1% to 20% yellow pea flour incorporation, averaging around 149 g [57].

Regarding porosity (Table 5), the common bean flour-enriched samples exhibited smaller pores, particularly with CB40. No significant differences were detected when increasing incorporation for all pulse species. No statistically significant differences were found when all samples were compared to the SC control.

The colorimetric parameters (Table 6) of the crust and crumb were assessed using the brown index, as bread tends to be brown during baking.

For the crust, the brown index ranged from 60.15 (GP40) to 40.62 (SC), with significant differences between CB and YP values compared to the control SC, except for GP20. The a* values ranged from 5.48 (CB40) to 14.26 (GP40), and b* values from 11.29 (CB40) to 27.52 (SC). The data indicate that the CB40 crust was less red than the control SC, while the YP and GP crusts exhibited higher a* values, reflecting a more reddish coloration. Differences in the individual chromatic coordinates, summarized by the ΔE parameter, confirmed these findings. The incorporation of pulse flours led to progressively greater color differences compared to the control. Increasing the level of grass pea flour resulted in enhanced browning, producing breads that appeared visibly more golden than the control.

Crumb color followed trends observed in the flours. The various pulse flour additions produced different colorations compared to both the control and among themselves, as confirmed by the ΔE parameter (Table 6). The brown index ranged from 64.45 (CB40) to 26.88 (SC), a* values from −2.05 (SC) to 2.92 (CB30), and b* values from 2.36 (CB40) to 27.03 (GP40). Breads enriched with common bean flour showed a darker crumb with a slightly pinkish hue, while those with grass pea and yellow pea flour exhibited a more yellowish tint, with higher b* values compared to the semolina-only control.

The incorporation of pulse flour influenced the texture of the bread (Table 7). Hardness ranged from 15.85 N (CB20) to 30.45 N (YP40), springiness from 0.84 (CB30) to 2.50 (GP40), gumminess from 59.16 N (CB20) to 244.41 N (CB40), chewiness from 60.98 N · mm (CB20) to 355.74 N · mm (GP40), and resilience from 0.30 (GP40) to 0.69 (YP20).

The effect of pulse flour on bread texture varied depending on the type and level of incorporation. While breads with higher percentages of yellow pea and grass pea flour tended to be harder, gummier, and exhibited greater chewiness, those with lower percentages of common bean flour (e.g., CB20) showed lower hardness and gumminess than the control. Springiness was slightly lower in the bean and yellow pea samples, although differences were not statistically significant, whereas higher grass pea flour incorporation resulted in significantly greater extensibility (2.50).

Regarding hardness, a similar trend has been reported in another study on the incorporation of yellow pea flour (1% to 20%), where hardness increased proportionally with the level of substitution [58].

**Figure 4:**
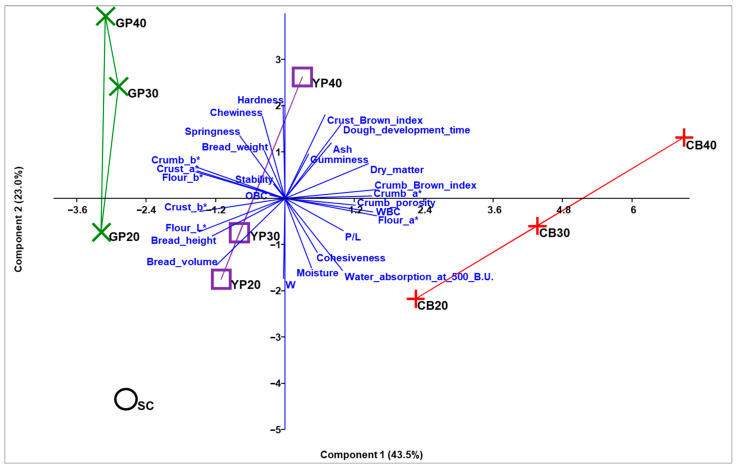
Principal component analysis (PCA) biplot. Vectors represent the loadings of the physical and chemical characteristics of flours, doughs, and breads obtained with different levels of integration of three pulse flours (at the following integration percentages: SC, semolina 100%; CB20, CB30, CB40 (common bean flour integration at 20%, 30%, and 40%, respectively); YP20, YP30, YP40 (yellow pea flour integration at 20%, 30%, and 40%, respectively); and GP20, GP30, GP40 (grass pea flour integration at 20%, 30%, and 40%, respectively). The groups integrated with the same pulse flour at different percentages are delimited by convex hulls.

As a result of the Principal Component Analysis (PCA), the first two components accounted for 66.4% of the total explained variance (PC1: 43.5%; PC2: 23.0%) (Figure 4), effectively distinguishing the flours, doughs, and breads obtained with increasing percentages of integration of three pulse flours (common bean, yellow pea and grass pea), in the multidimensional space (Table S1).

CB20, CB30, and CB40, along with YP40, were positioned in the positive region of PC1, exhibiting strong positive correlations (>70%)—and consequently high values—with flour (a*) and crumb (a*, brown index) color parameters, water-related properties of flour (WBC, dry matter), and crumb porosity. Conversely, SC, YP (20 and 30), and all GP samples (20, 30, and 40), located in the negative region of PC1, were associated with lower values for these parameters.

PC1 also displayed strong negative correlations (≥70%) with color parameters of the flour (L*, b*), crust (a*, b*), and crumb (b*), as well as with bread height and volume (Figure 3; Table S2).

All mixes with higher integration (GP40, YP40, CB40, and GP30) were positioned in the positive region of PC2, showing a strong positive correlation (>70%) with hardness, chewiness, crust brown index, and dough development time. PC2 also showed strong negative correlations (≥60%) with W, moisture and volume of bread. Consequently, SC, CB20, CB30, YP20, YP30, and GP20, located in the negative region of PC2, exhibited higher levels of these variables, in contrast with the mixes with higher concentrations (Figure 4; Table S2).

## 4. Conclusions

The study highlighted how the incorporation of legume flours into wheat flours significantly influences the physicochemical and technological properties of both the flours and the resulting breads. Formulations incorporating up to 30% yellow pea flour and grass pea flour produced breads with volumes slightly lower than the semolina-only control (SC = 276.25 cm^3^; YP30 = 251.25 cm^3^, GP30 = 246.25 cm^3^), although the differences were not statistically significant. These breads exhibited slightly denser porosity and higher values of dough stability and development. In particular, yellow pea flour at 30% demonstrated a good balance between dough workability and the characteristics of the final bread, suggesting its potential suitability for industrial bread production.

Common bean flour yielded the best results at 20%, with satisfactory volume and favorable texture parameters, particularly in terms of hardness, gumminess, and chewiness. However, common bean-based doughs were more tenacious and less capable of retaining fermentation gases, which could limit their application in leavened bread production. In comparison, yellow pea and grass pea doughs exhibited lower strength (W) and tenacity/extensibility ratio (P/L) than common bean doughs, which were stronger and more tenacious. This characteristic makes common bean flour more suitable for dry baked goods such as crackers and biscuits, where gas retention is not a primary requirement.

While PCA and other analyses indicate that 30% substitutions with yellow pea or grass pea flours produce breads with distinct characteristics compared to the control, these formulations still maintain acceptable technological properties. Therefore, the recommendation for industrial bread production should be considered carefully: 30% yellow pea flour appears promising due to its balanced dough and final bread properties, whereas grass pea flour at 30% may be suitable when the aim is to produce breads with modified texture and nutritional properties. Common bean flour is better suited at 20%, closely resembling control bread characteristics but with limitations for gas-retaining products.

Overall, the findings suggest that fortification with pulse flours represents a promising opportunity for the development of innovative baked products: sweet products such as biscuits, cookies, or rusks with common bean flour, and savory products such as crackers, breadsticks, or wraps with grass pea flour. Yellow pea flour offers potential for both flat breads and leavened products, although further optimization may be required to match the control bread characteristics fully.

## Figures and Tables

**Table 1:** Particle size distribution of pure and mixed flours.

	Particle Size Distribution (%)												
Ø (μm)	SC	CB	YP	GP	CB20	CB30	CB40	YP20	YP30	YP40	GB20	GB30	GB40
**>300**	0.1	6.2	6.0	2.2	0.8	0.7	1.0	0.5	0.1	0.4	1.2	0.8	1.2
**200–300**	0.5	35.0	29.0	6.8	1.2	1.3	1.5	0.8	0.9	1.6	0.8	1.2	1.8
**180–200**	31.4	47.2	51.0	29.0	44.0	45.0	46.5	40.7	38.0	40.0	30.0	26.0	27.0
**160–180**	48.0	9.3	8.0	41.0	35.0	38.0	31.0	42.0	40.6	39.7	43.2	52.7	56.8
**<160**	20.0	2.3	6.0	21.0	19.0	15.0	20.0	16.0	20.4	18.3	24.8	19.3	13.2

SC: control semolina; CB: semolina with common bean flour at different integration percentages (20/30/40%); YP: semolina with yellow pea flour at different integration percentages (20/30/40%); GP: semolina with grass pea flour at different integration percentages (20/30/40%).

**Table 2:** Formulation of the experimental breads (g/100 g of semolina).

Bread Type	Flour (g)	Bean (g)	Yellow Pea (g)	Grass Pea (g)	NaCl (g)	Water (g)
SC	100	-	-	-	2.2	62.70
CB20	80	20	-	-	2.2	63.95
CB30	70	30	-	-	2.2	64.10
CB40	60	40	-	-	2.2	64.25
YP20	80	-	20	-	2.2	63.80
YP30	70	-	30	-	2.2	63.50
YP40	60	-	40	-	2.2	61.55
GP20	80	-	-	20	2.2	62.15
GP30	70	-	-	30	2.2	59.50
GP40	60	-	-	40	2.2	56.15

SC: control semolina; CB: semolina with common bean flour at different integration percentages (20/30/40%); YP: semolina with yellow pea flour at different integration percentages (20/30/40%); GP: semolina with grass pea flour at different integration percentages (20/30/40%).

**Table 3:** Physical and technological characteristics of semolina, both on its own and when combined with pulse flours.

Sample	Dry Matter (g/100 g)	Ash (g/100 g d.m.)	L*	a*	b*	ΔE	WBC (g H_2_O/g d.m.)	OBC (g Oil/g d.m.)
SC	88.93 ± 0.12 c	0.79 ± 0.01 g	87.60 ± 0.55 ab	−1.91 ± 0.08 e	16.48 ± 0.20 f	0.0	1.73 ± 0.06 bc	2.76 ± 0.35
CB20	90.08 ± 0.45 abc	1.62 ± 0.02 d	85.82 ± 0.01 bcd	−1.50 ± 0.01 c	10.81 ± 0.03 g	6.0	1.92 ± 0.05 abc	2.44 ± 0.07
CB30	90.73 ± 0.23 ab	1.91 ± 0.04 c	84.48 ± 0.01 de	−1.05 ± 0.02 b	8.20 ± 0.01 h	8.9	2.06 ± 0.06 ab	2.61 ± 0.11
CB40	91.01 ± 0.06 a	2.29 ± 0.01 b	83.47 ± 0.07 e	−0.50 ± 0.03 a	5.41 ± 0.01 i	11.9	2.19 ± 0.02 a	2.54 ± 0.11
YP20	89.68 ± 0.06 bc	1.81 ± 0.01 c	87.76 ± 0.01 a	−1.82 ± 0.06 de	17.58 ± 0.01 e	1.1	1.74 ± 0.01 bc	2.41 ± 0.10
YP30	90.03 ± 0.01 abc	2.31 ± 0.02 b	85.79 ± 0.04 bcd	−1.74 ± 0.01 cde	18.65 ± 0.06 d	2.8	1.69 ± 0.05 bc	2.59 ± 0.13
YP40	90.58 ± 0.03 ab	2.87 ± 0.01 a	85.34 ± 0.63 cde	−1.65 ± 0.00 cd	19.30 ± 0.01 c	3.6	1.65 ± 0.06 c	2.45 ± 0.19
GP20	89.70 ± 0.14 bc	1.31 ± 0.01 f	87.79 ± 0.04 a	−2.46 ± 0.00 f	19.91 ± 0.01 b	3.5	1.71 ± 0.02 bc	2.37 ± 0.04
GP30	89.59 ± 0.13 bc	1.54 ± 0.01 e	87.03 ± 0.01 abc	−2.50 ± 0.02 f	20.97 ± 0.03 a	4.6	1.71 ± 0.04 bc	2.67 ± 0.01
GP40	89.53 ± 0.06 bc	1.68 ± 0.02 d	86.47 ± 0.03 abc	−2.48 ± 0.01 f	20.84 ± 0.00 a	4.5	1.65 ± 0.11 c	2.66 ± 0.12

SC: control semolina; CB: semolina with common bean flour at different integration percentages (20/30/40%); YP: semolina with yellow pea flour at different integration percentages (20/30/40%); GP: semolina with grass pea flour at different integration percentages (20/30/40%). The colorimetric parameters L*, a*, b*, and ΔE are dimensionless values. Different letters in a column indicate a significant difference: *p* ≤ 0.001 (Tukey); the absence of letters indicates absence of significance.

**Table 4:** Main rheological parameters of semolina and pulses mixed doughs: (data are means ± standard deviations).

	Farinograph			Alveograph	
Sample	Development Time (min)	Stability (min)	Water Absorption at 500 B.U. (%)	W (10^−4^ × J)	P/L
SC	1.80 ± 0.00 d	2.70 ± 0.28 bc	62.70 ± 0.1 ab	246.00 ± 8.49 a	5.25 ± 0.05 a
CB20	4.15 ± 0.64 bc	4.35 ± 0.64 ab	63.95 ± 0.49 a	167.50 ± 7.78 b	2.61 ± 0.17 c
CB30	4.50 ± 0.28 abc	3.05 ± 0.28 ab	64.10 ± 0.14 a	117.50 ± 3.54 c	3.49 ± 0.12 b
CB40	6.40 ± 0.00 a	3.90 ± 0.00 ab	64.25 ± 0.64 a	74.50 ± 4.95 de	5.91 ± 0.22 a
YP20	1.70 ± 0.14 d	4.00 ± 0.14 ab	63.80 ± 0.57 a	58.00 ± 1.41 fg	1.33 ± 0.00 d
YP30	3.30 ± 0.14 cd	3.05 ± 0.14 ab	63.50 ± 0.14 a	36.00 ± 0.00 gh	1.31 ± 0.08 d
YP40	4.60 ± 0.42 abc	1.25 ± 0.42 c	61.55 ± 0.64 ab	21.00 ± 0.00 h	2.43 ± 0.00 c
GP20	1.70 ± 0.14 d	3.95 ± 0.14 ab	62.15 ± 0.21 ab	101.00 ± 0.00 cd	1.47 ± 0.17 d
GP30	4.00 ± 0.42 bc	4.15 ± 0.42 ab	59.50 ± 1.98 bc	65.50 ± 0.71 e	1.19 ± 0.04 d
GP40	5.70 ± 0.00 ab	5.05 ± 0.00 a	56.15 ± 0.35 c	49.00 ± 0.00 fgh	1.41 ± 0.02 d

SC: control semolina; CB: semolina with common bean flour at different integration percentages (20/30/40%); YP: semolina with yellow pea flour at different integration percentages (20/30/40%); GP: semolina with grass pea flour at different integration percentages (20/30/40%). Different letters in a column indicate a significant difference (Tukey): *p* ≤ 0.001 (development time), *p* < 0.01 (water absorption at 500 B.U.) and *p* < 0.05 (stability). The P/L are dimensionless values.

**Table 5:** Physical properties of durum wheat bread at increasing level of replacement (20, 30, 40%) of re-milled semolina (control) prepared with flour of different pulses (data are means ± standard deviations).

Sample	Moisture (g/100 g)	Volume (cm^3^)	Height (mm)	Weight (g)	Porosity (1–8) *
SC	33.79 ± 0.40 ab	276.25 ± 1.77 a	55.25 ± 1.77 ab	152.70 ± 1.34 a	6.75 ± 0.14 ab
CB20	36.03 ± 0.30 a	250.00 ± 7.07 ab	52.95 ± 1.91 ab	154.44 ± 0.09 a	6.50 ± 0.01 ab
CB30	32.38 ± 0.88 ab	232.50 ± 7.07 bc	52.35 ± 1.34 ab	152.62 ± 0.17 a	7.00 ± 0.01 ab
CB40	33.20 ± 0.45 ab	208.75 ± 1.77 c	50.05 ± 1.91 b	152.75 ± 1.27 a	7.75 ± 0.05 a
YP20	34.01 ± 0.48 ab	243.75 ± 1.77 b	56.15 ± 1.34 a	149.25 ± 2.68 a	6.25 ± 0.01 ab
YP30	34.51 ± 0.59 ab	251.25 ± 5.30 ab	54.20 ± 0.71 ab	152.09 ± 4.35 a	5.75 ± 0.01 b
YP40	33.60 ± 0.08 ab	230.00 ± 3.54 bc	50.65 ± 1.48 ab	154.44 ± 1.20 a	6.00 ± 0.01 b
GP20	32.72 ± 0.26 ab	252.50 ± 7.07 ab	56.35 ± 0.07 a	155.12 ± 1.87 a	6.00 ± 0.03 b
GP30	31.59 ± 0.81 b	246.25 ± 1.77 ab	53.00 ± 0.71 ab	155.69 ± 0.83 a	6.00 ± 0.04 b
GP40	30.75 ± 0.57 b	238.75 ± 2.12 bc	55.50 ± 2.12 ab	154.36 ± 0.36 a	6.50 ± 0.02 ab

SC: control semolina; CB: semolina with common bean flour at different integration percentages (20/30/40%); YP: semolina with yellow pea flour at different integration percentages (20/30/40%); GP: semolina with grass pea flour at different integration percentages (20/30/40%). * Scale 1–8; 1 = non-uniform structure, large and irregular cells; 8 = uniform compact structure, small and regular cells. Different letters in a column indicate a significant difference (Tukey): *p* ≤ 0.001 (moisture, volume and porosity) and *p* < 0.05 (height and weight).

**Table 6:** Color parameters of re-milled semolina (control) and mixes: (data are means ± standard deviations).

	Crust				Crumb			
Sample	Brown Index (100 − L*)	a*	b*	ΔE	Brown Index (100 − L*)	a*	b*	ΔE
SC	40.62 ± 0.59 c	11.85 ± 0.25 ab	27.52 ± 0.34 a	0.0	26.88 ± 0.47 d	−2.05 ± 0.01 d	19.18 ± 0.16 d	0.0
CB20	54.69 ± 0.42 ab	7.80 ± 0.45 bc	13.76 ± 0.00 bcd	1.0	55.04 ± 1.12 b	2.59 ± 0.11 a	4.92 ± 0.04 e	0.7
CB30	57.11 ± 2.11 ab	6.08 ± 0.69 c	12.61 ± 0.91 cd	20.3	60.49 ± 1.48 ab	2.92 ± 0.23 a	4.24 ± 0.49 e	30.9
CB40	59.91 ± 2.35 a	5.48 ± 0.29 c	11.29 ± 0.56 d	19.7	64.45 ± 0.98 a	2.55 ± 0.2 a	2.36 ± 0.21 e	32.4
YP20	52.76 ± 1.80 ab	11.96 ± 0.54 a	21.21 ± 2.16 abc	22.4	38.38 ± 0.71 c	0.30 ± 0.33 bc	20.72 ± 0.11 cd	37.9
YP30	55.08 ± 1.66 ab	13.29 ± 0.04 a	23.11 ± 1.05 a	23.4	39.52 ± 0.67 c	0.80 ± 0.17 b	22.01 ± 0.39 bcd	35.8
YP40	55.82 ± 0.67 ab	13.31 ± 0.55 a	23.05 ± 1.36 a	26.8	38.17 ± 1.65 c	1.05 ± 0.04 b	22.55 ± 0.28 bcd	41.8
GP20	48.58 ± 0.74 bc	13.09 ± 0.76 a	24.14 ± 2.26 a	25.0	33.36 ± 0.18 cd	−1.44 ± 0.08 d	23.46 ± 0.98 abc	40.5
GP30	58.75 ± 1.53 a	13.96 ± 1.05 a	21.81 ± 1.14 ab	11.6	32.77 ± 0.52 cd	−1.42 ± 0.20 d	24.63 ± 1.19 ab	12.0
GP40	60.15 ± 0.98 a	14.26 ± 0.52 a	21.43 ± 0.00 ab	15.3	38.48 ± 1.78 c	−0.78 ± 0.19 cd	27.03 ± 0.00 a	11.0

SC: control semolina; CB: semolina with common bean flour at different integration percentages (20/30/40%); YP: semolina with yellow pea flour at different integration percentages (20/30/40%); GP: semolina with grass pea flour at different integration percentages (20/30/40%). Different letters in a column indicate a significant difference (Tukey): *p* ≤ 0.001. The colorimetric parameters Brown index, a*, b*, and ΔE are dimensionless values.

**Table 7:** Texture profile analysis (TPA) of breads with increasing levels of different pulse flours (20%, 30%, 40%): (data are means ± standard deviations).

Sample	Hardness (N)	Springness	Gumminess (N)	Chewiness (N · mm)	Resilience
SC	16.10 ± 2.69 c	1.11 ± 0.14 c	126.41 ± 1.54 e	130.60 ± 2.05 g	0.64 ± 0.01 ab
CB20	15.85 ± 2.62 c	1.00 ± 0.01 c	59.16 ± 2.73 g	60.98 ± 1.05 i	0.37 ± 0.01 ab
CB30	20.25 ± 0.21 bc	0.84 ± 0.01 c	124.18 ± 0.64 e	102.79 ± 0.35 h	0.52 ± 0.24 ab
CB40	24.00 ± 0.42 abc	0.98 ± 0.05 c	244.41 ± 4.79 a	234.15 ± 1.40 c	0.67 ± 0.00 a
YP20	16.40 ± 0.14 c	0.98 ± 0.01 c	181.14 ± 0.94 c	180.00 ± 2.85 e	0.69 ± 0.02 a
YP30	21.70 ± 0.14 abc	0.96 ± 0.1 c	83.66 ± 0.67 f	81.24 ± 2.79 i	0.63 ± 0.14 ab
YP40	30.45 ± 0.92 a	0.92 ± 0.01 c	217.42 ± 1.34 b	198.24 ± 0.55 d	0.39 ± 0.04 ab
GP20	21.10 ± 1.98 abc	1.00 ± 0.03 c	160.85 ± 3.70 d	159.66 ± 0.60 f	0.36 ± 0.03 ab
GP30	26.85 ± 0.64 ab	1.58 ± 0.04 b	159.78 ± 1.87 d	252.95 ± 0.49 b	0.39 ± 0.01 ab
GP40	25.15 ± 0.78 abc	2.50 ± 0.02 a	130.29 ± 1.03 e	325.74 ± 0.52 a	0.30 ± 0.04 b

SC: control semolina; CB: semolina with common bean flour at different integration percentages (20/30/40%); YP: semolina with yellow pea flour at different integration percentages (20/30/40%); GP: semolina with grass pea flour at different integration percentages (20/30/40%). Different letters in a column indicate a significant difference (Tukey): *p* ≤ 0.001 and *p* < 0.01 (only resilience).

## Data Availability

The original contributions presented in the study are included in the article/Supplementary Materials; further inquiries can be directed to the corresponding authors.

## Supplementary Materials

The following supporting information can be downloaded at: https://www.mdpi.com/article/10.3390/foods14213720/s1, Table S1: Principal Component Analysis (PCA). Eigenvalue and proportion of variance explained by each principal component; Table S2: Principal component analysis (PCA) loadings of the physical and chemical characteristics of flours, doughs, and breads obtained with different levels of integration of three legumes flour (bean, pea, and grass pea).
