# Supplementary material for: Qualitative Characteristics of Semolina–Pulse Flour Mixes and Related Breads
Source: Foods. 2025 Oct 30;14(21):3720. doi: 10.3390/foods14213720 (PMC12609456; doi:10.3390/foods14213720)
Supplement: Supplementary file 1 [file foods-14-03720-s001.zip › foods-3918582-supplementary.pdf]

Table S1. Principal Component Analysis (PCA). Eigenvalue and proportion of variance explained by each principal component.

PC	Eigenvalue	% variance	Cumulative variance %
1	12,1649	43,446	43,446
2	6,43429	22,98	66,426
3	3,378	12,064	
4	2,29829	8,2082	
5	1,75443	6,2658	
6	1,11877	3,9956	
7	0,426917	1,5247	
8	0,287818	1,0279	
9	0,136564	0,48773	

Table S2. Principal component analysis (PCA) loadings of the physical and chemical characteristics of flours, doughs, and breads obtained with different levels of integration of three legumes flour (bean, pea, and grass pea), at the following integration percentages: SC, semolina 100%; CB20, CB30, CB40 (bean flour integration at 20%, 30%, and 40%, respectively); YP20, YP30, YP40 (pea flour integration at 20%, 30%, and 40%, respectively); GP20, GP30, GP40 (grass pea flour integration at 20%, 30%, and 40%, respectively).

	Axis 1	Axis 2	Axis 3	Axis 4	Axis 5	Axis 6	Axis 7	Axis 8	Axis 9	Axis 10
**Dry matter (g/100 g)**	0,8744	0,3299	-0,3097	-0,04623	-0,05504	-0,06025	-0,116	-0,06197	0,06776	1,87E-14
**Ash (g/100 g d.m.)**	0,4859	0,5282	-0,6531	0,03738	0,002858	0,1987	0,1118	-0,06276	0,03258	-3,58E-15
**Flour_L***	-0,8971	-0,3176	0,01103	-0,04994	0,1835	-0,1945	0,009622	0,03532	-0,1375	-2,02E-15
**Flour_a***	0,9607	-0,1642	-0,06809	0,1744	0,02777	0,09254	0,06172	-0,03114	0,03037	-2,21E-15
**Flour_b***	-0,9352	0,2501	-0,2451	0,0154	-0,00378	0,04271	0,01904	0,01599	-0,00534	2,12E-15
**WBC (g H2O/d.m.)**	0,9204	-0,1284	0,3011	-0,01128	0,07317	-0,1185	-0,1344	0,08608	0,02616	-1,51E-15
**OBC (g oil/d.m.)**	-0,1633	0,03068	0,6012	0,4025	-0,2416	0,5954	-0,1576	0,0931	-0,05055	9,64E-16
**Dough development time (min)**	0,586	0,7014	0,2692	-0,0357	-0,1532	0,1831	0,178	-0,00748	0,04634	-1,29E-15
**Stability (min)**	-0,1211	0,1364	0,6188	-0,5325	0,4752	-0,1036	0,0708	0,2213	0,09842	1,38E-15
**Water absorption at 500 B.U. (%)**	0,6031	-0,6842	-0,3623	0,01924	0,0623	-0,0726	-0,1022	0,1265	0,02924	-1,48E-15
**W (10-4 x J)**	-0,01176	-0,7618	0,5211	0,0766	-0,3369	-0,114	0,07081	-0,05546	-0,08688	4,63E-15
**P/L**	0,6119	-0,3096	0,375	0,5598	-0,1907	-0,0757	0,1563	-0,02498	0,09282	-1,35E-15
**Moisture (g/100 g)**	0,2784	-0,6629	-0,432	-0,2445	-0,1381	0,05972	0,4348	0,158	-0,00543	2,55E-15
**Crust_Brown index (100-L*)**	0,4207	0,7956	-0,0006	-0,3162	0,1617	0,1923	-0,04787	0,1287	-0,08919	8,80E-16
**Crust_a***	-0,9198	0,263	-0,2449	0,08147	-0,00092	0,09592	0,07275	0,03952	0,04565	1,41E-15
**Crust_b***	-0,8919	-0,124	-0,2111	0,3456	-0,09178	0,04606	0,05445	-0,02925	0,1036	1,98E-16
**Crumb_Brown index (100-L*)**	0,9475	0,08236	0,1238	-0,2661	0,06404	0,01043	-0,0353	-0,0553	0,02799	-3,58E-15
**Crumb_a***	0,9068	0,02455	-0,1935	-0,3117	-0,01445	0,1393	0,04056	-0,1263	-0,073	-1,46E-15
**Crumb_b***	-0,9138	0,2917	-0,2078	0,1454	0,08513	0,06724	0,0142	-0,01286	0,0578	-1,17E-16
**Bread volume (cm3)**	-0,7167	-0,6342	0,1087	-0,05179	-0,2291	0,1283	0,001049	0,02344	-0,01424	3,73E-15
**Bread height (mm)**	-0,7617	-0,3603	0,1501	-0,2064	0,4	-0,02832	-0,0725	-0,1881	0,1533	-1,33E-15
**Bread weight (g)**	-0,2159	0,4532	0,2276	-0,2047	-0,7043	-0,3421	-0,05041	0,1694	0,1002	-1,58E-15
**Crumb porosity (1-8)***	0,7376	-0,06519	0,5682	0,2929	0,1231	-0,1017	0,08361	-0,0975	0,03185	-8,11E-16
**Hardness (N)**	-0,02121	0,8891	-0,2366	0,2658	-0,2658	-0,02961	-0,06895	0,07116	0,03196	-3,68E-15

**Springness**	-0,4737	0,5918	0,5699	-0,1085	0,1101	0,1896	0,1829	-0,08509	0,00455	2,35E-15
**Gumminess (N)**	0,2476	0,422	-0,1856	0,6521	0,2784	-0,4632	0,03794	0,04824	-0,07049	7,96E-16
**Chewiness (N x mm)**	-0,2343	0,7793	0,3318	0,3113	0,2897	-0,1513	0,1377	0,0129	-0,06948	3,45E-15
**Cohesiveness**	0,3389	-0,5161	-0,1358	0,4924	0,4659	0,3097	-0,03368	0,2017	0,05597	1,56E-15
